# Strategies and Applications of Graphene and Its Derivatives-Based Electrochemical Sensors in Cancer Diagnosis

**DOI:** 10.3390/molecules28186719

**Published:** 2023-09-20

**Authors:** Li Fu, Yuhong Zheng, Xingxing Li, Xiaozhu Liu, Cheng-Te Lin, Hassan Karimi-Maleh

**Affiliations:** 1 Key Laboratory of Novel Materials for Sensor of Zhejiang Province, College of Materials and Environmental Engineering, Hangzhou Dianzi University, Hangzhou 310018, China; lixingxing@hdu.edu.cn; 2 Jiangsu Key Laboratory for the Research and Utilization of Plant Resources, Institute of Botany, Jiangsu Province & Chinese Academy of Sciences (Nanjing Botanical Garden Mem. Sun Yat-Sen), Nanjing 210014, China; 3 Department of Critical Care Medicine, Beijing Shijitan Hospital, Capital Medical University, Beijing 100054, China; xiaozhuliu2021@163.com; 4 Qianwan Institute, Ningbo Institute of Materials Technology and Engineering (NIMTE), Chinese Academy of Sciences, Ningbo 315201, China; linzhengde@nimte.ac.cn; 5 Center of Materials Science and Optoelectronics Engineering, University of Chinese Academy of Sciences, Beijing 100049, China; 6 Key Laboratory of Marine Materials and Related Technologies, Zhejiang Key Laboratory of Marine Materials and Protective Technologies, Ningbo Institute of Materials Technology and Engineering (NIMTE), Chinese Academy of Sciences, Ningbo 315201, China; 7 School of Resources and Environment, University of Electronic Science and Technology of China, Chengdu 611731, China; hassan@uestc.edu.cn; 8 School of Engineering, Lebanese American University, Byblos 1102-2801, Lebanon

**Keywords:** graphene, electrochemical sensor, cancer biomarker, early diagnosis, nanocomposite, biorecognition, microfluidics, point of care

## Abstract

Graphene is an emerging nanomaterial increasingly being used in electrochemical biosensing applications owing to its high surface area, excellent conductivity, ease of functionalization, and superior electrocatalytic properties compared to other carbon-based electrodes and nanomaterials, enabling faster electron transfer kinetics and higher sensitivity. Graphene electrochemical biosensors may have the potential to enable the rapid, sensitive, and low-cost detection of cancer biomarkers. This paper reviews early-stage research and proof-of-concept studies on the development of graphene electrochemical biosensors for potential future cancer diagnostic applications. Various graphene synthesis methods are outlined along with common functionalization approaches using polymers, biomolecules, nanomaterials, and synthetic chemistry to facilitate the immobilization of recognition elements and improve performance. Major sensor configurations including graphene field-effect transistors, graphene modified electrodes and nanocomposites, and 3D graphene networks are highlighted along with their principles of operation, advantages, and biosensing capabilities. Strategies for the immobilization of biorecognition elements like antibodies, aptamers, peptides, and DNA/RNA probes onto graphene platforms to impart target specificity are summarized. The use of nanomaterial labels, hybrid nanocomposites with graphene, and chemical modification for signal enhancement are also discussed. Examples are provided to illustrate applications for the sensitive electrochemical detection of a broad range of cancer biomarkers including proteins, circulating tumor cells, DNA mutations, non-coding RNAs like miRNA, metabolites, and glycoproteins. Current challenges and future opportunities are elucidated to guide ongoing efforts towards transitioning graphene biosensors from promising research lab tools into mainstream clinical practice. Continued research addressing issues with reproducibility, stability, selectivity, integration, clinical validation, and regulatory approval could enable wider adoption. Overall, graphene electrochemical biosensors present powerful and versatile platforms for cancer diagnosis at the point of care.

## 1. Introduction

Cancer remains one of the leading causes of mortality worldwide, responsible for nearly 10 million deaths in 2020 alone [1]. The early diagnosis and treatment of cancer is critical to improving patient survival rates. However, conventional cancer diagnosis techniques such as imaging, biopsy, and laboratory testing have limitations including invasiveness, high cost, low sensitivity, and delays in obtaining results [2]. There is an urgent need for simple, rapid, and ultrasensitive analytical techniques that allow early cancer detection and real-time monitoring of disease progression and treatment response.

In recent years, electrochemical biosensors have emerged as promising tools for point-of-care cancer diagnostics owing to their high sensitivity, selectivity, rapid response, low sample volume requirement, and capability for integration into portable devices [3,4,5,6]. Electrochemical biosensors convert a biological recognition event into a measurable electronic signal through an electrochemical transducer [7,8,9,10]. They rely on the use of biomolecules like antibodies, aptamers, and peptides or DNA as biorecognition elements to provide specificity towards target cancer biomarkers [11,12]. Transduction is achieved by monitoring the current, potential, or impedance changes resulting from redox reactions or the binding events at electrode interfaces functionalized with the biorecognition element [13,14].

Graphene, a single layer of sp^2^-bonded carbon atoms arranged in a honeycomb lattice, has recently catalyzed research interest for electrochemical sensors due to its exceptional properties, including high surface area, excellent electrical conductivity, good mechanical strength, ease of functionalization, and excellent biocompatibility [15,16]. The high surface-to-volume ratio allows efficient immobilization of biorecognition elements, while excellent conductivity facilitates rapid electron transfer for sensitive electrochemical measurement [17,18]. Additionally, graphene demonstrates excellent electrocatalytic activity, allowing lower overpotentials and better selectivity [19]. These properties make graphene an ideal material to enhance the performance of electrochemical biosensors.

Various forms of graphene such as graphene oxide (GO), reduced graphene oxide (rGO), graphene quantum dots, and three-dimensional graphene structures have been explored for electrochemical cancer biosensing [20]. For instance, GO provides more functional groups for the covalent attachment of biomolecules while rGO offers higher conductivity [21]. Three-dimensional graphene structures such as hydrogels and foams allow greater loadings of biorecognition elements owing to their high surface area [22]. Graphene composites with noble metals, metal oxides, and conducting polymers can further improve electrocatalysis and biocompatibility [23].

Surface functionalization strategies play a key role in fabricating effective graphene electrochemical biosensors [24]. Biorecognition elements such as antibodies, DNA probes, aptamers, and peptides need to be stably immobilized on the sensor surface while retaining bioactivity. Non-covalent approaches based on π–π stacking interactions are simple but result in random orientation and low stability. Covalent immobilization via reactive functional groups on GO produces stable linkages oriented perpendicular to the surface [25]. Graphene field-effect transistors allow for the label-free electrical detection of cancer biomarkers through changes in surface charge or potential [26,27]. Signal enhancement strategies such as enzyme amplification and nanomaterial labels have been coupled with graphene electrochemical biosensors to further lower detection limits and meet clinical requirements [28,29].

Graphene electrochemical biosensors have been applied for the sensitive detection of a variety of cancer biomarkers. For protein biomarker detection, graphene-based electrochemical immunosensors have been developed for the sensitive detection of common cancer proteins like prostate specific antigen (PSA) [30], carcinoembryonic antigen (CEA) [31], and cancer antigen 125 (CA125) in clinical samples [32]. Graphene-modified electrochemical genosensors have demonstrated capabilities for detecting cancer-related DNA mutations such as in BRAF [33], KRAS [34], and EGFR [35] genes in liquid biopsy and patient tissue samples [36]. Aptamer-based graphene biosensors have shown promise in the detection of cancer biomarkers like vascular endothelial growth factor (VEGF) [37] and thrombin [38] down to picomolar levels. Advances have also been made in using graphene materials to modify electrodes for the sensitive quantification of circulating tumor cells [39], allowing the rapid analysis of blood samples. Detection limits down to femtomolar levels have been achieved along with high selectivity against other interfering species. Graphene-modified electrodes, nanocomposite materials, and three-dimensional structures have all demonstrated excellent performance as electrochemical biosensor platforms. Real sample analysis has also been performed to establish the clinical validity of the sensors [40]. This review summarizes recent advances in the fabrication strategies used to create graphene electrochemical biosensors and their applications specifically in cancer diagnosis. The following sections highlight graphene synthesis and modification approaches, different sensor configurations, immobilization strategies for biorecognition elements, transduction mechanisms, and finally, examples of application towards the detection of key cancer biomarkers and cells. Current challenges and future opportunities towards point-of-care cancer diagnostic devices based on graphene electrochemical biosensor technology are discussed.

## 2. The Synthesis and Modification of Graphene Materials

Graphene can be produced via a range of bottom-up and top-down approaches, each with their own advantages and limitations in terms of quality, scalability, and cost. The physical and chemical properties of graphene depend strongly on the synthesis method, since defects, contaminants, and functional groups introduced during production can alter graphene’s electrochemical performance and biosensing capabilities. Additionally, surface functionalization is often required to improve graphene’s solubility, prevent aggregation, and allow the covalent immobilization of biorecognition elements. This section summarizes the major fabrication strategies for graphene along with key functionalization approaches to enable biosensing applications.

### 2.1. Methods for Graphene Synthesis

**Figure 1:**
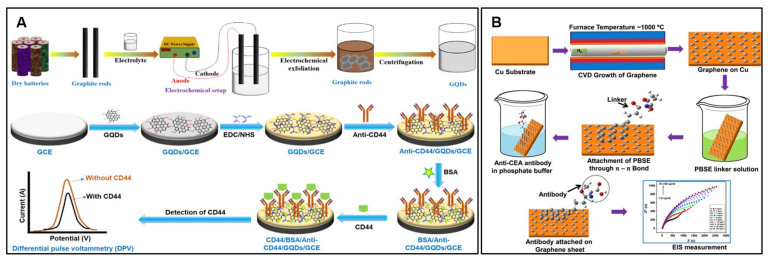
(**A**) Schematic diagram of electrochemically exfoliated GQDs and the sensing mechanism of the fabricated electrochemical biosensor. (**B**) Schematic of CVD-based biosensor fabrication and EIS measurements. Reproduced with permission [44,47].

Mechanical exfoliation, based on repeatedly peeling flakes from graphite using adhesive tape, produced the first isolated graphene sheets and remains widely used for fundamental studies [41]. It generates pristine, high-quality graphene, but the process is time-consuming, has low yield, and is unsuitable for scaling up [42]. Liquid-phase exfoliation increases productivity by ultrasonically cleaving graphite in suitable solvents like N-methylpyrrolidone, although this mostly produces multilayer graphenes [43]. Electrochemical exfoliation applies a bias voltage to induce oxidation and intercalation between graphite layers, facilitating their separation in solution. For example, electrochemical exfoliation plays a pivotal role in enhancing the performance of the electrochemical biosensor for the specific detection of CD44 breast cancer biomarkers [44]. This technique yields graphene quantum dots (GQDs) with remarkable properties that directly translate to improved sensing capabilities (Figure 1A). The GQDs produced through electrochemical exfoliation exhibit uniform size, high electrochemical conductivity, and abundant surface functional groups, which collectively contribute to the sensor’s enhanced performance. The high conductivity of GQDs facilitates efficient electron transfer between the GQD-modified electrode and the CD44 biomarker, resulting in a strong and distinguishable signal response. Additionally, the availability of numerous active sites on the GQD surface enables effective immobilization of CD44 antibodies, ensuring selective binding and accurate detection of the target analyte. This method’s ability to generate GQDs with well-defined properties contributes to the stability and reproducibility of the sensor, leading to consistent and reliable performance.

Chemical vapor deposition (CVD) allows for the large-area synthesis of high-quality monolayer graphene by decomposing hydrocarbons on transition metal substrates like Cu and Ni [45]. Graphene can then be transferred to other surfaces via the wet etching of the metal foil. While scalable, CVD graphene has grain boundaries, wrinkles, and substrate-induced doping effects [46]. For example, CVD-derived graphene serves as a pivotal element in the functioning of this biosensor, playing a crucial role in enhancing its sensitivity and specificity. For this biosensing platform, graphene is grown on a Cu substrate through CVD, resulting in the formation of large, uniform, continuous, and atomically thin graphene films (Figure 1B) [47]. The unique structural and electrical properties of CVD-grown graphene provide an exceptional platform for biofunctionalization. In the context of cancer biomarker detection, the biosensor is designed to detect carcinoembryonic antigen (CEA), a protein associated with several types of cancer. The graphene surface is functionalized with 1-pyrenebutanoic acid succinimidyl ester, creating a specific binding site for the covalent immobilization of anti-CEA antibodies. This graphene-based electrode, modified with anti-CEA antibodies, facilitates the selective and sensitive detection of CEA through electrochemical impedance spectroscopy (EIS) measurements. The CVD-grown graphene provides a high specific surface area and excellent electrical conductivity, offering an ideal substrate for antibody immobilization and ensuring efficient electron transfer. The sensitivity achieved by this biosensor is remarkable, with a linear response in the physiological range of CEA concentrations, showcasing a sensitivity of 563.4 Ω ng/mL/cm^2^, a correlation coefficient of 0.996, and a low limit of detection (LOD) of 0.23 ng/mL.

Epitaxial growth produces pristine graphene directly on silicon carbide wafers but requires ultrahigh temperatures (>1100 °C), limiting its applications. For example, Tehrani et al. [48] reported a biosensor that used epitaxial graphene grown on SiC substrates. Epitaxial graphene were produced on large-area SiC wafers using standard semiconductor processing, allowing for the scalable fabrication of graphene-based devices. The graphene surface was functionalized with phenyl amine groups using an electrochemical diazotization process. This allowed antibodies to be attached to the graphene surface. The microchannels of the functionalized epitaxial graphene were used as the sensing element. The high surface area of graphene allowed for the sensitive detection of binding events on the channel surface. The analyte detected was 8-hydroxydeoxyguanosine (8-OHdG), which is a biomarker associated with oxidative stress and cancer risk. The binding of the 8-OHdG biomarkers to antibodies on the graphene surface caused detectable changes in the electrical conductivity across the channels. This allowed sensitive, label-free electrochemical detection. The epitaxial graphene biosensor could detect 8-OHdG at concentrations as low as 0.1 ng/mL, which is five times lower than the detection limits of standard ELISA immunoassay tests.

In contrast, the reduction of GO prepared by oxidizing and exfoliating graphite via Hummers’ method or a modified Hummers’ method is a simple, low-cost approach [49]. However, residual oxygen content reduces electrical conductivity compared to pristine graphene [50]. For example, Rostamabadi and Heydari-Bafrooei [51] developed an electrochemical aptasensor for detecting the breast cancer biomarker HER2. The sensor used a GCE modified with a nanocomposite made of electrochemically reduced graphene oxide (ErGO), SWCNTs, and AuNPs. The anti-HER2 aptamer was then immobilized onto this nanocomposite surface. ErGO was utilized in this sensor because it improves conductivity and provides a large surface area for aptamer immobilization. The performance of the aptasensor was analyzed by EIS. The aptamer specifically binds to HER2, increasing the charge transfer resistance. This allows for the sensitive detection of HER2 down to 50 fg/mL, with a wide analytical range from 0.1 pg/mL to 1 ng/mL. Shafiei et al. [52] proposed an electrochemical aptasensor using rGO-chitosan-AuNPs composite for detecting breast cancer cells. The aptamer AS1411, which binds to overexpressed nucleolin protein on cancer cells, was used as the biosensor recognition element. The researchers found that modifying the sensor surface with the composite significantly improved performance for detecting MCF-7 breast cancer cells. The GO provided a large surface area and high conductivity. Chitosan offered biocompatibility and sites for immobilizing the aptamer. AuNPs enabled strong aptamer binding and further enhanced conductivity. Together, the synergistic effects of the composite components greatly increased the sensitivity and selectivity. When cancer cells bound to the aptamer, this blocked the electron transfer to the sensor surface, allowing detection by EIS. The aptasensor showed high selectivity for cancer cells over normal cells, a wide detection range spanning from 10^1^ to 10^6^ cells/mL, and a low limit of detection of just 4 cells/mL. The rGO was a key factor enabling ultrasensitive detection by increasing the electrical conductivity and effective surface area of the sensor. This demonstrates the potential of graphene-based aptasensors for early cancer diagnosis.

### 2.2. Functionalization Strategies for Biosensing

**Figure 2:**
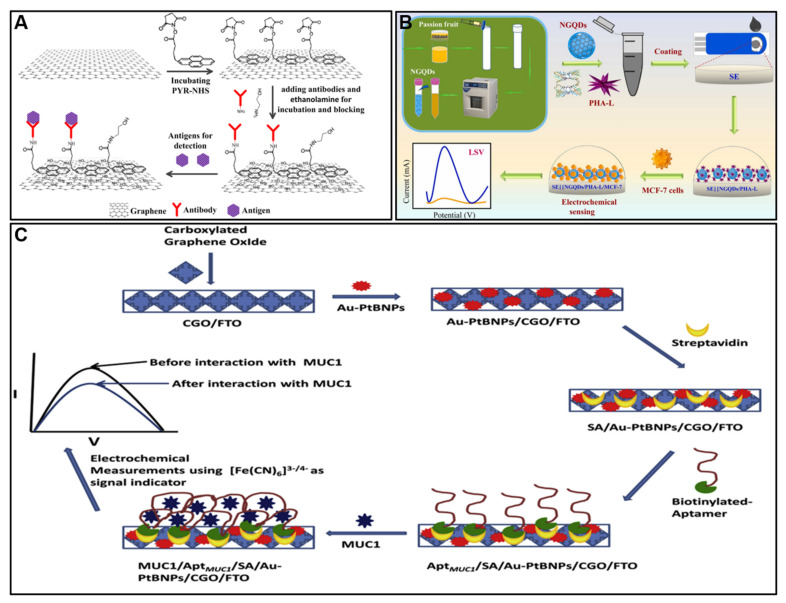
(**A**) A schematic diagram of all the modification steps for GFET. Reproduced with permission. (**B**) Synthesis of NGQDs from passion fruit juice using the microwave method for the electrochemical detection of MCF-7 cancer cell lines. (**C**) Fabrication of electrochemical aptasensor for MUC1 detection using Au-PtBNPs/CGO. Reproduced with permission [53,58,60].

The sp^2^-hybridized carbon lattice provides graphene chemical inertness and hydrophobicity, which poses challenges for biosensing applications. Surface functionalization is often required to make graphene hydrophilic, prevent aggregation in the solution, and enable the stable immobilization of biomolecules. Non-covalent approaches utilize physical adsorption or π–π stacking interactions but provide random orientation and moderate stability. For example, a bifunctional linker molecule, 1-pyrenebutanoic acid succinimidyl ester (PYR-NHS), was utilized to interact with the graphene surface via non-covalent π-stacking interactions [53]. This interaction between PYR-NHS and graphene forms a stable interface that does not disrupt the intrinsic properties of graphene. Subsequently, the succinimide ester group of PYR-NHS selectively reacts with the amine groups on anti-CEA antibodies, facilitating their immobilization onto the graphene surface (Figure 2A). This immobilization creates a platform where the anti-CEA antibodies are strategically positioned to interact with CEA molecules. When CEA molecules bind to the immobilized antibodies, the electrical properties of the graphene channel are modified, leading to changes in the drain–source current (Ids) of the graphene field-effect transistor (GFET). The specific binding of CEA to the anti-CEA modified GFET results in a real-time increase in drain current, enabling the highly sensitive and specific detection of CEA proteins. This non-covalent modification strategy not only preserves the integrity of the graphene material but also enhances the sensor’s ability to selectively capture target molecules, making it a promising tool for clinical applications in cancer diagnostics and other biomolecular detection scenarios.

In contrast, covalent linking via reactive oxygen groups creates oriented monolayers ideal for biorecognition. GO contains epoxy, hydroxyl, and carboxyl groups that enable a wide range of covalent biomolecule conjugation approaches. Carbodiimide coupling is commonly used to activate carboxyl groups on GO for amidation with amine-containing biomolecules. For example, carbodiimides like EDC coupled with NHS facilitate the covalent attachment of proteins or DNA via amide bond formation [54]. Controlled reaction conditions prevent excessive grafting so that the biomolecules remain accessible for recognition. Amine-epoxides readily form under mild conditions to tether amino-modified oligonucleotides [55]. This provides an efficient approach to tethering amino-modified oligonucleotides or other amine-containing biomolecules. By tuning the reaction parameters, the grafting density can be optimized to balance covalent attachment with the retention of accessibility. Diazonium grafting produces robust aryl linkages on pristine graphene [56]. Pyrene and related aromatic linkers allow for π stacking [57].

Doping graphene with foreign atoms is an effective approach to tune properties like electrical conductivity and electrocatalytic activity. Nitrogen doping improves hydrophilicity and reactivity besides creating defects that enhance electrochemical behavior [19]. For example, Tran et al. [58] utilized N-doped graphene quantum dots (NGQDs) as the nanocarrier for the lectin phytohemagglutinin-L (PHA-L), which specifically binds to breast cancer cells. The NGQDs were synthesized from passion fruit juice via a microwave-assisted hydrothermal method, resulting in inherent nitrogen doping in the graphene structure (Figure 2B). The nitrogen doping enhances the electrical conductivity and electrocatalytic activity of the NGQDs. When the NGQDs are deposited on the screen-printed electrode, the high conductivity provides excellent electronic interactions between the electrode and NGQDs, increasing the electroactive sites and mass transfer rate. This results in a remarkably low charge transfer resistance of 5.5 Ω for the NGQD-modified electrode, compared to 890 Ω for the bare electrode. The NGQDs serve as an efficient electronic mediator to enhance the current response for detection of breast cancer cells. The ultrasensitive electrochemical sensor modified with NGQD/PHA-L exhibited a wide linear detection range of 5–10^6^ cells/mL in phosphate-buffered saline and 20–10^6^ cells/mL in human serum, with low detection limits of 1 and 2 cells/mL, respectively.

Depositing noble metals like gold and platinum generates nanocomposites with excellent electrocatalysis and high surface area [21]. Elshafey et al. [59] reported the use of Au-NPs-decorated graphene for the electrochemical immunosensing of p53 antibodies. The immunosensor uses Au NPs assembled on graphene oxide (GO) films on screen-printed carbon electrodes. The Au NPs provide a large surface area for immobilizing the p53 antigen to capture anti-p53 antibodies. The p53 antigen is attached to the Au NPs through electrostatic interactions. This helps maintain the bioactivity of the p53 antigen for antibody binding. The sensor detects the binding of anti-p53 antibodies using a label-free approach by monitoring the changes in the redox peak current of a [Fe(CN)_6_]^3−/4−^ probe. The Au NPs contribute to the high sensitivity of the immunosensor. Bharti et al. [60] reported an electrochemical aptasensor that utilizes gold–platinum bimetallic nanoparticles (Au-PtBNPs) deposited on carboxylated graphene oxide (CGO) nanosheets for the detection of mucin 1 (MUC1) protein. The synergistic effect of both Au and Pt NPs showed enhanced electrochemical properties compared to the mono-metallic nanoparticles. Pt NPs helped improve the electrical conductivity and electrocatalytic activity of the CGO electrode surface. The Au-PtBNPs/CGO nanocomposite provided a suitable immobilization platform for the aptamer to capture MUC1 protein (Figure 2C). This aptasensor exhibits high sensitivity with a detection limit of 0.79 fM and wide linear range from 1 fM to 100 nM for MUC1 detection. The bimetallic Au-Pt nanoparticles play a key role in signal enhancement and improving the overall analytical performance of the aptasensor for MUC1 detection.

**Figure 3:**
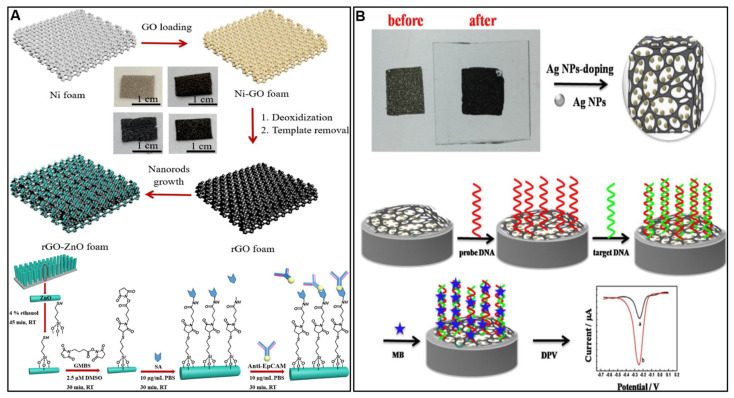
(**A**) Schematic diagram of the preparation process of the self-supporting rGO-ZnO foam and biological modification. (**B**) Photographs of the fabrication process for 3D GF/AgNPs alongside the DNA biosensor construction scheme. Reproduced with permission [61,62].

Three-dimensional graphene foams, sponges, and hydrogels provide enormous surface area for biofunctionalization. Crosslinking GO with small molecules or polymers produces porous networks ideal for loading high quantities of nanomaterials and biomolecules. Su et al. [61] described the development of a 3D graphene macroporous structure modified with ZnO nanorods and antibodies (rGO-ZnO-antiEpCAM) that can selectively capture CTCs (Figure 3A). The large pore structure allows efficient CTC adhesion deep into the 3D matrix. Modification with ZnO nanorods provides sites for antibody attachment and enhances cell–substrate interactions. Anti-EpCAM antibodies enable specific binding to epithelial CTCs. When CTCs bind to the structure, the electrical properties of the conductive graphene are affected. Resistance measurements showed that the rGO-ZnO-antiEpCAM structure’s resistance increased over time as more CTCs adhered, likely due to negative charges of the cells interfering with conduction in p-type graphene. Electrochemical impedance spectroscopy also revealed increasing charge transfer resistance during CTC capture, indicating that the cells hindered charge transfer at the electrode surface. Overall, the graphene macroporous structure enabled selective CTC adhesion, while electrical measurements provided real-time monitoring of cell binding through measurable changes in resistance and impedance. Chen et al. [62] reported a three-dimensional electrochemical DNA biosensor that was developed for the sensitive detection of CYFRA21-1 DNA, a marker associated with non-small-cell lung cancer (NSCLC). The biosensor utilized a composite of 3D graphene functionalized with Ag nanoparticles, enhancing probe ssDNA immobilization and electron transfer efficiency (Figure 3B). Under optimal conditions, the biosensor demonstrated high sensitivity, detecting target DNA down to 1.0 × 10^−14^ M with a linear range spanning from 1.0 × 10^−14^ to 1.0 × 10^−7^ M. Clinical trials successfully detected CYFRA21-1 DNA in real lung cancer samples, showcasing its potential for early lung cancer diagnosis.

Overall, the diversity of oxygen groups on GO coupled with the ability for metal doping and 3D assembly allow for extensive modifications to improve graphene’s performance in electrochemical biosensing.

## 3. Graphene Electrochemical Biosensors

### 3.1. Different Sensor Designs and Fabrication Strategies

Various designs for electrochemical biosensors that incorporate graphene materials as the transducer have been explored for sensitive and selective cancer diagnosis. These utilize different sensor fabrication strategies including GFET, graphene-modified electrodes, nanocomposites with graphene, and 3D graphene networks. Each configuration offers specific advantages arising from graphene’s unique properties. This section highlights these major graphene electrochemical biosensor platforms along with their preparation, operating principles, and performance advantages.

Graphene is an ideal transducer material for FETs due to its exceptional charge carrier mobilities allowing rapid current modulation [63]. Graphene FETs (GFETs) modulate current flow between source and drain electrodes by altering carrier density and type in the semimetallic graphene channel using a gate voltage [64]. In GFET biosensors, the specific binding of charged target biomolecules to the graphene or to the attached receptors alters the local potential distribution. This causes changes in drain current proportional to analyte concentration without need for labels or redox probes [65]. GFETs provide direct, real-time electrical readouts of binding events and are easily integrated with microfluidics for portable operation [66]. High sensitivity down to femtomolar target detection is enabled by graphene’s low electrical noise and ability for effective gating modulation [67].

Graphene can be incorporated into FETs through various fabrication strategies. Large-area graphene synthesized by CVD allows wafer-scale production but requires transfer onto insulating substrates like SiO_2_. For example, a sensor may use CVD-grown graphene as the transducing element in a FET biosensor for detecting the cancer biomarker HER3 [68]. The graphene provides high electrical conductivity and carrier mobility, allowing for the sensitive electrical detection of binding events on the surface. The FET is then functionalized with PtNPs, which provide sites for immobilizing HER3-specific antibodies. When HER3 antigens bind to the antibody receptors on the surface, it causes a change in the electrical characteristics of the graphene FET, allowing sensitive and specific detection of HER3 down to concentrations of 300 fg/mL. The high-quality monolayer graphene grown by CVD, combined with surface functionalization using PtNPs and HER3 antibodies, allows the creation of a highly sensitive and selective biosensor for detecting this important cancer biomarker.

**Figure 4:**
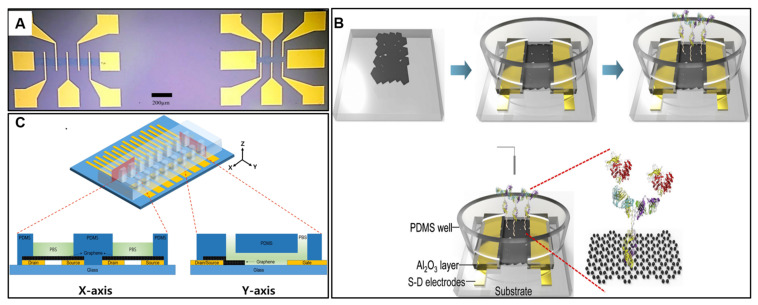
(**A**) GFET devices with asymmetric (left) and symmetric (right) voltage sense electrodes. Graphene channels, in the central regions of the FET devices, have a dark blue contrast compared to the Si/SiO_2_ substrate. (**B**) Schematic of the R-GO FET fabrication and detection of the PSA-ACT complex. (**C**) Schematic of the microchannel integrated into solution-gated GFET with the coplanar electrode array. Reproduced with permission [67,69,76].

Alternatively, graphene produced by the reduction of GO can be deposited from solution, while exfoliation and inkjet printing produce smaller sheets. Gold source and drain electrodes with a spacing of a few microns are patterned by photolithography or electron beam lithography. For example, through lithographic patterning, precise and controlled graphene channels can be fabricated on a Si/SiO_2_ substrate, forming the basis of the sensor (Figure 4A) [69]. This patterning process allows for the creation of well-defined, nanoscale structures that can interact with the targeted analyte. The lithography technique enables the selective deposition of chromium and gold electrodes, which serve as the source, drain, and voltage sense electrodes, forming the electronic connections for electrical measurements. Additionally, lithography facilitates the controlled attachment of linker molecules and biomolecules onto the graphene surface, crucial for achieving the desired functionalization and selectivity of the sensor. This functionalization involves the successive attachment of a linker molecule (Pyr-NHS ester), anti-hCG antibodies, and blocking agents (bovine serum albumin), ultimately leading to the specific binding of the hCG antigen. The binding reaction between the immobilized anti-hCG antibodies and hCG antigen generates changes in the electrical properties of the graphene channel, resulting in measurable variations in resistance.

Pristine graphene provides ambipolar transfer characteristics, while unintentional doping necessitates asymmetric metal contacts [70,71]. The choice of dielectric layer impacts the gating control and sensitivity. The functionalization of the graphene channel is key to impart selectivity. Antibodies [32], aptamers [72], peptides [73], and DNA probes [74] have been covalently tethered for specific cancer biomarker recognition. Graphene’s high transconductance and low electrical noise boosts sensitivity for the detection of charged biomolecules. GFETs have demonstrated an ability to detect cancer biomarkers like prostate-specific antigens and the prostate-specific antigen/α1-antichymotrypsin (PSA-ACT) complex [67]. The reduced graphene oxide FETs (R-GO FETs) were fabricated through a self-assembly process, and immunoreactions between PSA-ACT complexes and immobilized PSA monoclonal antibodies on the R-GO channel surface induced shifts in the gate voltage, specifically at the point of minimum conductivity (Vg, min) (Figure 4B). This shift exhibited a linear relationship with the analyte concentration, enabling detection down to femtomolar levels (100 fg/mL or ∼1.1 fM) with a dynamic range spanning six orders of magnitude. High association constants of 3.2 nM^−1^ and 4.2 nM^−1^ were achieved for different pH conditions. The biosensor demonstrated specificity to the target PSA-ACT complex in both phosphate-buffered saline solutions and human serum.

Multiplexing is readily achieved by fabricating arrays of GFET sensors within a small chip [75]. For example, multiplexing plays a crucial role in enhancing the capabilities of the developed solution-gated GFET sensor for TP53 DNA detection [76]. Multiplexing refers to the simultaneous detection of multiple analytes within a single device, allowing for the parallel measurement of different targets (Figure 4C). The coplanar electrode array integrated with the microfluidic chip facilitates the immobilization of different probe DNA sequences specific to different DNA targets on separate electrode pairs. This arrangement allows the sensor to perform multiplexed DNA detection, where each electrode pair can independently capture and monitor the hybridization of complementary, mismatched, and noncomplementary DNA samples. By detecting multiple DNA sequences simultaneously, the sensor gains versatility, improves throughput, and conserves sample volume. This multiplexing capability enhances the efficiency of cancer diagnostics by providing a comprehensive analysis of multiple DNA targets within the same experiment, ultimately contributing to a more comprehensive and accurate assessment of the biological samples under investigation. Microfluidic integration allows portable operation with low sample consumption. However, fluid gate dielectrics suffer from drift effects and nonspecific adsorption. Biofunctionalization can also dampen carrier mobility. Overall, GFETs are promising electronic biosensor platforms which harness graphene’s excellent transistor behavior for sensitive and rapid cancer diagnosis.

Graphene nanosheets strongly adsorbed or covalently tethered onto conductive electrode surfaces serve as immobilization scaffolds for biorecognition elements in electrochemical biosensors [77]. Graphene materials promote electron transfer between the redox-active portions of surface-bound biomolecules and the electrode [78]. Their high surface area increases the loading of recognition elements to amplify sensor signals. GO in particular provides abundant oxygen functional groups for covalent bioconjugation. RGO offers higher conductivity, which is beneficial for electrocatalysis [51]. Graphene coatings retain biomolecule activity and minimize surface fouling effects. For example, Wang et al. [79] developed an antifouling electrochemical DNA biosensor for detecting the breast cancer marker BRCA1, using a nanocomposite of the conducting polymer PEDOT doped with a zwitterionic polypeptide. The PEDOT/polypeptide composite forms a 3D porous nanostructure, providing a large surface area and excellent hydrophilicity for DNA probe attachment while resisting nonspecific protein adsorption. The biosensor demonstrates high sensitivity with a detection limit of 0.0034 pM BRCA1, as well as good selectivity against mismatches and interference species. Importantly, the biosensor retains performance in 1% human serum, exhibiting negligible fouling from serum proteins. This is attributed to the zwitterionic polypeptide dopant, which confers excellent antifouling ability.

Additionally, graphene’s excellent electrocatalytic behavior improves selectivity against interfering molecules. For example, Ruiyi et al. [80] reported a novel electrochemical sensor for detecting cancer cells, based on a hybrid nanomaterial made of folic acid and glutamic-acid-functionalized graphene quantum dots combined with a palladium-gold core-shell nanostructure (FA/Glu-GQD-Pd@Au). The authors found that the FA/Glu-GQD component provided targeting ability and electrochemical activity, while the Pd@Au core-shell structure exhibited excellent electrocatalytic activity to amplify detection signals. The sensor leveraged the synergistic effects of the hybrid nanomaterial, offering ultrahigh sensitivity down to 2 cells/mL with a wide detection range of 3 to 105 cells/mL. Testing in blood samples showed good recovery rates of 95.1–102.6%. The key innovation was using the electrocatalytic properties of the Pd@Au core-shell structure to greatly enhance detection signals. This enabled ultrasensitive electrochemical detection through the redox activity of the FA/Glu-GQD component.

Solution processing allows for the straightforward modification of various electrode materials with graphene nanosheets. Inkjet printing [35], electrophoretic deposition [81], and dropcasting [82] produce uniform graphene coatings from suspensions. Electrodeposition and CVD provide thinner and higher quality films. Pyrolytic methods form graphene directly from carbon precursors. The binding of aromatic mediators assists strong π–π stacking on electrodes [83]. The immobilization of biorecognition elements onto graphene occurs through physical adsorption or covalent linkages [84]. Target cancer biomarkers are detected through changes in the electrode current, potential, or impedance.

Nanocomposite materials incorporating graphene with noble metal nanoparticles, metal oxides, and other nanostructures combine the advantages of each component to synergistically enhance biosensing performance. Relative to bare graphene, nanocomposites demonstrate increased electrocatalytic activity, higher conductivity, improved biocompatibility, and larger available surface area for the immobilization of biomolecules. Graphene aids the dispersion of the nanomaterials and enables efficient charge transfer. The nanomaterials facilitate electrochemical reactions at lower overpotentials and provide numerous active sites for the binding of recognition elements. Additionally, some metal nanoparticles can serve as electroactive labels for signal amplification.

**Figure 5:**
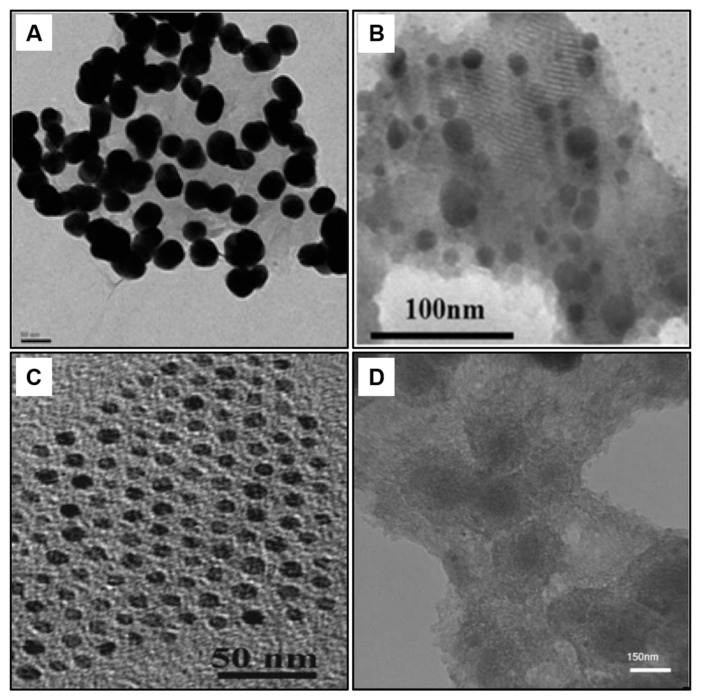
Morphology of several graphene-based nanocomposites for constructing electrochemical sensors for cancer diagnosis. (**A**) Graphene–gold composite, (**B**) graphene–silver composite, (**C**) graphene–iron oxide composite, (**D**) CuO/WO_3_–GO composite. Reproduced with permission [86,87,88,89].

Facile wet chemical synthesis produces colloidal dispersions of various nanoparticle–graphene composites [85]. Common combinations utilized in electrochemical biosensors include graphene–gold [86], graphene–platinum [60], graphene–silver [87], graphene–iron oxide [88] and graphene–metal oxides like CuO and TiO_2_ [81,89]. Hydrothermal approaches [85], electrostatic assembly [90], electrophoretic deposition [91], and electrochemical reduction methods [51] allow for nanoscale control over morphology and composition. Figure 5 shows the morphology of several graphene-based nanocomposites. Nanomaterials introduce functional groups like amines to improve bioconjugation and biocompatibility. Proteins, antibodies, aptamers, and DNA are effectively immobilized on the high-surface-area composites.

**Figure 6:**
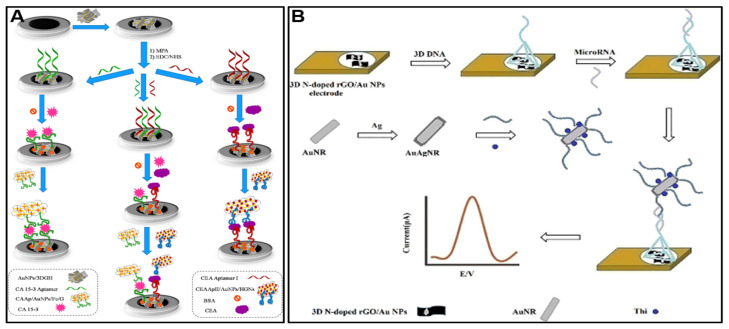
Morphology of several graphene-based nanocomposites for constructing electrochemical sensors for cancer diagnosis. (**A**) Schematic diagram of the fabrication of 3D graphene hydrogel nanocomposites to simultaneously detect CEA and CA 15-3. (**B**) Schematic diagram of the fabrication of 3D N-doped rGO/AuNPs for miRNA-155 detection. Reproduced with permission [92,95].

Three-dimensional graphene materials, including foams, sponges, hydrogels, and aerogels, overcome the tendency for nanosheets to aggregate, while providing extremely high surface-area-to-volume ratios. This allows higher loading of biorecognition elements and efficient electrocatalysis in the 3D graphene scaffold. Three-dimensional networks maintain graphene’s electrical conductivity and excellent mechanical properties. The open, porous structure facilitates the rapid mass transport of analytes to the electrode surface. The high surface area amplifies signals, enabling the detection of cancer biomarkers at extremely low concentrations. For example, Shekari et al. [92] proposed a dual electrochemical aptasensor using 3D graphene hydrogel nanocomposites to simultaneously detect CEA and CA 15-3. The aptasensor uses gold nanoparticles loaded with 3D graphene hydrogel (AuNPs/3DGH) as a sensing substrate, providing a large surface area and high conductivity to enhance detection sensitivity (Figure 6A). Redox probes labeled aptamers specific for CEA and CA 15-3 provide electrochemical signals for each biomarker. The aptasensor demonstrated wide linear detection ranges of 5.0 × 10^−2^–60 ng/mL for CEA and 5.0 × 10^−2^–100 U/mL for CA 15-3, with low detection limits of 11.2 pg/mL for CEA and 1.3 × 10^−2^ U/mL for CA 15-3. Testing in spiked human serum samples showed good recovery and accuracy compared to ELISA.

Various chemical and physical approaches produce 3D graphene architectures. The self-assembly of GO driven by strong π–π stacking and hydrophobic interactions forms hydrogels that can be dried into aerogels [93,94]. Chemical reduction yields partially restored electrical conductivity. Template synthesis produces 3D graphene conformal to sacrificial scaffolds [62]. Doping with heteroatoms like nitrogen further tunes properties. For example, Tian et al. [95] described the development of an ultrasensitive electrochemical biosensor for detecting miRNA-155 based on a 3D nitrogen-doped reduced graphene oxide/gold nanoparticle (3D N-doped rGO/AuNPs) composite electrode (Figure 6B). The 3D N-doped rGO provided a high surface area substrate to immobilize a DNA tetrahedral structure probe for capturing the target miRNA-155. The binding of miRNA-155 resulted in the attachment of a gold–silver nanorod tag labeled with a redox-active thionine reporter. The 3D graphene electrode gave a wide dynamic range of 1 × 10^−11^ to 1 × 10^−4^ M and a low limit of detection of 1 × 10^−12^ M miRNA-155. The high sensitivity can be attributed to the large surface area and excellent conductivity of the 3D N-doped rGO, which enhanced the immobilization of the DNA probes and electron transfer. The biosensor showed high selectivity against non-target sequences and good recovery for miRNA-155 detection in serum samples.

These 3D graphene materials exhibit higher sensitivity by at least one order of magnitude compared to 2D structures. Electrocatalysis and electron transfer kinetics are greatly enhanced. Stability remains a key challenge. Overall, 3D graphene architectures represent attractive scaffolds for ultrasensitive electrochemical cancer biosensing.

### 3.2. Immobilization of Biorecognition Elements

The biorecognition layer imparts specificity towards the target cancer biomarkers in electrochemical biosensors. Immobilization strategies play a critical role in fabricating sensors with high sensitivity, selectivity, stability, and reproducibility. Physical adsorption and covalent conjugation are widely employed to attach model biorecognition elements like antibodies, aptamers, peptides, and DNA/RNA probes onto graphene platforms. The orientation, surface density, and bioactivity retention of the immobilized biomolecules determine sensor performance and clinical applicability.

Antibodies offer high specificity and affinity towards cancer protein biomarkers through molecular recognition. Their immobilization onto graphene electrochemical biosensors occurs via physisorption that utilizes hydrophobic and electrostatic interactions or through covalent linkage chemistries to oxygen functional groups [96,97]. Physisorption is simple and preserves antibody structure but can cause random orientation and weak attachment. Covalent binding produces stable and ordered monolayers ideal for sensitive and reproducible detection. The 1-Ethyl-3-(3-dimethylaminopropyl)carbodiimide (EDC) coupling of carboxyl groups on GO to primary amines on antibodies is widely employed [98,99]. Multistep carbodiimide-mediated conjugation protocols with additional reagents like NHS improve efficiency [100]. Amine-epoxide addition reactions under mild conditions also covalently tether antibodies to GO [101]. Pyrene and related π-stacking groups non-covalently immobilize antibodies in oriented configurations [102]. Diazonium grafting produces robust covalent linkage but can damage biomolecules [103]. Blocking agents like BSA minimize nonspecific adsorption.

Aptamers are single-stranded oligonucleotides selected in vitro to bind specific molecular targets with high affinity and selectivity with regard to rivaling antibodies. Their thermal and chemical stability, ease of modification, and reproducible synthesis make them appealing biorecognition elements for electrochemical biosensors. Aptamers are immobilized on graphene electrodes through π–π stacking interactions and covalent attachment using standard coupling reagents [104]. Carbodiimide chemistry using EDC/NHS activates carboxyl groups on GO for covalent aptamer linkage [105]. Copper-catalyzed azide–alkyne cycloaddition (CuAAC) efficiently attaches aptamers bearing terminal alkynes [106]. Aptamers exhibit enhanced stability over antibodies with comparable sensitivities. Their small size provides high surface coverage and improved electron transfer. Modifications like locked nucleic acids and the incorporation of fluorophores further augment selectivity and lower detection limits [107]. Aptamers matched to cancer biomarkers enable specific identification in complex clinical samples.

Immobilized single-stranded DNA or RNA probes on graphene transduce hybridization events with complementary nucleic acid targets into measurable electrical signals for sensitive and selective cancer diagnosis. Physical adsorption based on π–π stacking between nucleotide bases and graphene sp^2^ lattice is simple but produces randomly oriented strands. For example, the capture probe DNA (DNA-c) with an amino group at the 5’ end is immobilized directly onto a graphene-modified GCE through π–π stacking interactions between the DNA bases and graphene surface [108]. The target DNA (DNA-t) is then hybridized to the immobilized capture probe, leaving the other half of the target strand available for hybridization. The reporter probe DNA (DNA-r) conjugated to gold nanoparticles is then hybridized to the remaining half of the target DNA strand. The oxidation peak current of gold nanoparticles increases proportionally with target DNA concentration, allowing sensitive electrochemical detection. The sensor can detect target DNA down to one femtomolar concentration with high selectivity. Covalent attachment using amide or epoxide linkages to the probes containing terminal amines better controls surface coverage and orientation. For example, the covalent attachment of DNA probes to a polypyrrole–graphene composite film was critical for the high performance of this electrochemical DNA biosensor [109]. It enabled the stable, oriented immobilization of probes at high density, facilitating efficient hybridization and electron transfer. Click chemistry provides robust linkages while retaining probe bioactivity. For example, An et al. [110] reported an electrochemical aptasensor for detecting tumor exosomes based on click chemistry and hybridization chain reaction (HCR) for signal amplification. The sensor uses CD63 aptamer to capture exosomes nonspecifically on the electrode surface. Then, alkynyl-4-ONE, a functionalized lipid electrophile, is conjugated to the exosomes via click chemistry between its alkyne group and azide on the DNA probe anchor. This allows for the sensitive detection of exosomes without targeting specific surface proteins. The HCR of DNA probes on the anchor amplifies the signal. The sensor detected exosomes in concentrations ranging from 1.12 × 10^2^ to 1.12 × 10^8^ particles/μL, with a limit of detection of 96 particles/μL. The key advantages of this biosensor were the use of click chemistry for the efficient nonspecific labeling of exosomes and HCR signal amplification to enhance sensitivity.

Electrostatic attraction aids in the immobilization of negatively charged DNA but requires positively charged surfaces. Pyrene [111], poly-L-lysine [112], and other cationic modifiers promote attachment. Packaging ssDNA into nanoparticles before immobilization improves stability [113,114]. Short probes enhance hybridization kinetics while minimizing nonspecific adsorption. Immobilized DNA or RNA probes enable the specific identification of cancer genomic biomarkers.

## 4. Applications in Cancer Diagnosis

Graphene-based electrochemical biosensors have been widely explored for the sensitive and selective detection of a variety of cancer biomarkers, including proteins, DNA, CTCs, miRNAs, and metabolites. Early diagnosis and screening to identify these biomarkers can significantly improve clinical outcomes for cancer patients. This section highlights the various applications of graphene electrochemical sensors for the analysis of different cancer biomarkers.

### 4.1. Detection of Cancer Protein Biomarkers

**Figure 7:**
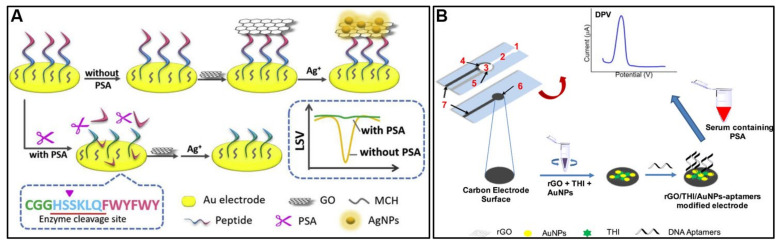
(**A**) Illustration of the peptide-cleavage-based electrochemical biosensor for the detection of PSA. (**B**) Fabrication and modification process of the microfluidic-paper-based aptasensor and the typical response regarding the detection of PSA. Reproduced with permission [30,115].

A number of protein biomarkers are extensively used in cancer diagnosis, prognosis, and the evaluation of treatment efficacy. PSA is the most commonly used biomarker for early prostate cancer screening. Meng et al. [115] reported an electrochemical biosensor for detecting PSA using peptide cleavage and graphene oxide/silver nanoparticle composites (Figure 7A). The sensor utilizes a PSA-specific peptide immobilized on a gold electrode. In the presence of PSA, the peptide is cleaved, which prevents the subsequent binding of graphene oxide and silver nanoparticle formation. This change in nanoparticle formation, measured by linear sweep voltammetry, allows for the ultrasensitive detection of PSA down to 0.33 pg/mL. The biosensor demonstrated a wide linear detection range of 5 pg/mL to 20 ng/mL PSA. It showed high selectivity against other proteins like AFP and CEA. An analysis of spiked serum samples demonstrated good recovery of between 83.4 and 106.7%. Zhang et al. [116] reported an electrochemical aptasensor for the detection of PSA. The aptasensor uses hemin-functionalized graphene conjugated to palladium nanoparticles (H-Gr/PdNPs) as a nanomaterial interface on a GCE. PSA aptamers are immobilized on the surface using a biotin-streptavidin system. In the presence of PSA, some of the aptamers are released, resulting in increased electron transfer and stronger electrochemical signals. The aptasensor demonstrated a broad linear detection range of 0.025–204.8 ng/mL for PSA, with a low limit of detection of 8 pg/mL. It showed good selectivity against other proteins and a recovery rate of 95–100% for PSA spiked in serum samples. Wei et al. [30] reported an electrochemical aptamer sensor that uses a paper microfluidic device for the sensitive detection of PSA (Figure 7B). The device uses screen-printed paper electrodes modified with gold nanoparticles, reduced graphene oxide, and thionine as the electrochemical transducer. A DNA aptamer targeting PSA is immobilized on the electrode surface. When PSA binds to the aptamer, it causes a change in the electrochemical signal from the thionine mediator that is proportional to the PSA concentration. The sensor can detect PSA down to 10 pg/mL with a detection range of 0.05 to 200 ng/mL. Testing with clinical serum samples showed good agreement with a reference hospital lab method. Table 1 details recent graphene-based electrochemical sensors for PSA detection.

**Figure 8:**
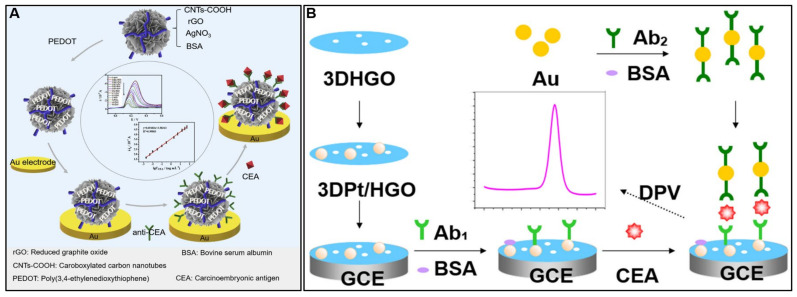
(**A**) Schematic representation of the construction of the CNTs-COOH/rGO/Ag@BSA/PEDOT. (**B**) Schematic illustration of the fabrication process of CEA/BSA/Ab_1_/3DPt/HGO-MGCE. Reproduced with permission [31,123].

CEA is an important clinical biomarker for colon, breast, lung, and pancreatic cancer diagnosis and disease monitoring. A new electrochemical immunosensor for the detection of CEA was reported by [123]. The immunosensor uses a nanocomposite made of carboxylated carbon nanotubes, reduced graphene oxide, silver–bovine serum albumin hybrid nanoflowers, and poly(3,4-ethylenedioxythiophene) deposited on a gold electrode (Figure 8A). Anti-CEA antibodies are immobilized on the nanocomposite. The unique structure and composition of the nanocomposite provide excellent electrochemical activity, high conductivity, large surface area, and stability. The immunosensor showed a linear detection range for CEA of 0.002–50 ng/mL with a low detection limit of 0.0001 ng/mL. It also demonstrated good selectivity, reproducibility, and stability. Testing in spiked human serum samples resulted in 93–102% recovery and it agreed well with commercial ELISA kits. Jing et al. [31] described an electrochemical immunosensor using a composite of three-dimensional, porous, GO-supported PtNPs (3DPt/HGO) to modify a GCE (Figure 8B). This composite provides a large surface area and good conductivity to immobilize and capture antibodies and amplify signals. AuNPs labeled with horseradish peroxidase and secondary antibodies were used to further amplify the detection signal. The immunosensor demonstrated a wide linear detection range of 0.001–150 ng/mL for CEA and a low limit of detection of 0.0006 ng/mL. It showed good selectivity against interferents; reproducibility, with a 5.1% RSD; and stability over 2 weeks. Spike-recovery experiments in human serum demonstrated 94.2–107% recovery. Table 2 details recent graphene-based electrochemical sensors for CEA detection.

In addition to protein biomarkers, aberrant glycan patterns serve as signatures for cancer progression and metastasis. Graphene-oxide-based electrochemical biosensors have been developed for detecting cancer-associated glycoproteins. Huang et al. [131] reported an electrochemical sensor for detecting the glycoprotein ovalbumin (OVA). The sensor uses a composite made of boronate-modified RGO and molecularly imprinted polymers (BGR@MIP) as the recognition element. The boronate groups bind specifically to the cis-diol groups on OVA, while the imprinting creates cavities that match the size and shape of OVA. This combination allows for the highly selective detection of OVA. The sensor was prepared by attaching boronic acid groups to graphene oxide, imprinting OVA on this modified graphene surface using sol-gel polymerization, and then removing the OVA. The resulting imprinted cavities could rebind to OVA. Tests showed that the sensor had excellent selectivity for OVA over other proteins. It could detect OVA at concentrations as low as 2 × 10^−11^ mg/mL, with a wide linear detection range of 10^−10^ to 10^−4^ mg/mL OVA. Klukova et al. [132] described the development of an ultrasensitive electrochemical biosensor using GO to detect glycoproteins down to attomolar concentrations. The biosensor uses the lectin Concanavalin A (Con A) directly immobilized on a GO-modified GCE, without any polymer modifier. This direct immobilization of Con A on GO allows for the sensitive, label-free detection of glycoproteins using EIS. The biosensor can detect the glycoprotein invertase (INV) with a limit of detection better than 1 aM and a wide linear detection range spanning seven orders of magnitude. Its sensitivity is 8.46 ± 0.20% per decade. The study also showed that the oxidative debris on GO improves the binding of Con A compared to graphene oxide that has been base-washed (GObw) to remove oxidative debris. The GO-based biosensor has about twice the sensitivity of the GObw-based sensor.

### 4.2. The Detection of Circulating Tumor Cells

**Figure 9:**
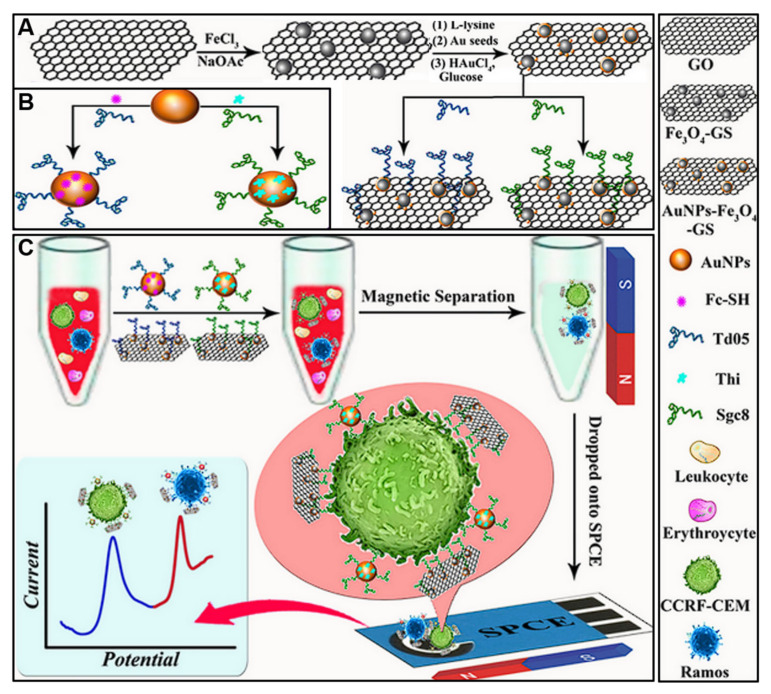
Illustration of the synthesis of (**A**) the aptamer-functionalized AuNPs-Fe_3_O_4_-GS capture probes and (**B**) the aptamer/electroactive-species-loaded AuNP amplification signal probes and (**C**) the capture, isolation, and amplified, multiplexed detection of the target CTCs in whole blood. Reproduced with permission [39].

CTCs are cancer cells shed from solid tumors into the bloodstream, serving as an emerging biomarker for cancer diagnosis and prognosis. The isolation and detection of rare CTCs among billions of blood cells remains technically challenging. GO substrates have been explored with regard to the sensitive capture and electrochemical detection of CTCs, leveraging the enhanced adhesion of CTCs onto graphene surfaces [39]. The aptamer-immobilized GO allows for the specific capture of target CTCs, while the gold nanoparticles amplify the signal (Figure 9). Using two different aptamers with distinct electroactive species, the sensor can simultaneously detect two types of CTCs based on their distinct oxidation potentials. The sensor showed a wide detection range of 5–500 cells/mL and low detection limits of 4 cells/mL for Ramos cells and 3 cells/mL for CCRF-CEM cells. It also demonstrated specificity against other cancer cells and the ability to detect spiked CTCs in whole blood. The simultaneous and sensitive detection of multiple CTC types could allow convenient cancer diagnosis from minimally invasive blood samples. Sadeghi et al. [133] proposed an electrochemical biosensor for detecting HER2-positive breast cancer cells. The biosensor uses a hybrid nanocomposite material made of reduced graphene oxide nanosheets and rhodium nanoparticles deposited on a graphite electrode. This nanomaterial provides a large surface area and high conductivity, allowing for the sensitive detection of cancer cells. The biosensor is functionalized with an anti-HER2 aptamer that specifically binds to HER2 receptors on cancer cells. When tested with HER2-positive SKBR3 cells, the biosensor demonstrated a wide linear detection range of 5 to 100,000 cells/mL and an ultra-low limit of detection of just 1 cell/mL. The biosensor showed excellent selectivity in distinguishing HER2-positive cancer cells from other cell types. Table 3 details recent graphene-based electrochemical sensors for CTCs detection.

### 4.3. DNA-Based Cancer Detection

Nucleic acid biomarkers like mutations, single nucleotide polymorphisms, altered gene expression, and methylation patterns provide valuable tools for cancer diagnosis. Cai et al. [140] fabricated a graphene and gold nanoparticle nanocomposite biosensor for the electrochemical detection of BRCA1 gene mutation, an important early indicator for breast and ovarian cancer risk. The researchers designed a novel label, SBA-15 coated with BMIM·BF4, and combined it with functionalized graphene and enzymes. The sandwich-type immunoassay demonstrated exceptional sensitivity, with a wide working range (0.01–15 ng/mL) and an impressively low detection limit of 4.86 pg/mL for BRCA1. This ultrasensitive detection ability was attributed to the synergistic effects of the label, which retained enzyme bioactivity and enhanced electron transport. In another work, a highly sensitive electrochemical genosensor was developed using a RGO-yttria nanocomposite (rGO:Y) as the immobilization platform for detecting the breast-cancer-related BRCA1 gene [141]. The sensor utilized a sandwich assay approach, with a capture probe DNA immobilized on a glassy carbon electrode modified with rGO:Y, and a AuNPs cluster-labeled reporter DNA for signal amplification. The rGO:Y nanocomposite provided enhanced sensitivity due to its high surface area and efficient electron transfer properties. The sensor exhibited remarkable sensitivity, with a linear detection range spanning from 10 aM to 1 nM DNA target concentrations and a detection limit of 5.95 aM. Işın et al. [142] used a pencil graphite electrode modified with a GO and ionic liquid composite (GO-IL-PGE) as the sensing platform. The detection was based on the direct voltammetric measurement of guanine oxidation after the hybridization of a BRCA1 probe DNA with complementary target BRCA1 DNA. Experimental conditions like probe concentration and hybridization time were optimized. The sensor showed a limit of detection of 1.48 μg/mL BRCA1 DNA and could discriminate between fully complementary and mismatched target sequences. The disposable GO-IL-PGE biosensor provides a simple, rapid way to detect BRCA1 DNA in 35 min. Xia et al. [143] constructed a biosensor based on a composite of 3D-rGO and PANI nanofibers modified on a GCE. The 3D porous structure of the RGO provides a high surface area for immobilizing DNA probes, while the PANI improves conductivity and electrochemical activity. The DNA probes specifically bind to complementary BRCA1 target sequences. Using differential pulse voltammetry, the biosensor demonstrated a wide detection range of 1 × 10^−15^ to 1 × 10^−7^ M BRCA1, with a very low limit of detection of 3.01 × 10^−16^ M.

The TP53 gene, also known as the tumor protein 53 or p53, is one of the most well-known tumor suppressor genes in the human body. Its role is critical in preventing the development and progression of cancer. Mutations in the TP53 gene are implicated in a wide range of cancers, and its importance in cancer diagnosis and understanding is substantial. Avila et al. [144] developed a DNA biosensor using screen-printed carbon electrodes modified with RGO-carboxymethylcellulose hybrid nanomaterials for detecting mutations in the TP53 tumor suppressor gene. The sensors utilize DNA hairpin probes of two different lengths to detect synthetic target DNA sequences, achieving detection limits around 3 nM without amplification. Using a 33-nucleotide probe provided complete discrimination between fully matched and single-nucleotide mismatched DNA targets. The sensor could directly detect a 100 nM synthetic target in untreated complex biological fluids like serum and saliva. Finally, it was able to successfully discriminate between cDNAs from breast cancer cell lines with wildtype and mutant TP53 genes.

The KRAS gene (Kirsten rat sarcoma viral oncogene homolog) is a critical gene that codes for a protein involved in cell signaling pathways. Mutations in the KRAS gene are commonly associated with various types of cancers, particularly colorectal, lung, and pancreatic cancers. The presence of KRAS mutations is important for cancer diagnosis and treatment. Al-Ogaidi et al. [34] used modified graphite and graphene pastes to detect the KRAS genetic marker for colon cancer in whole blood samples. The sensors were modified with a phthalocyanine-BODIPY dye or azulene dyes to improve performance. The sensor with the phthalocyanine-BODIPY dye and graphite had a limit of quantification of 1.54 × 10^−4^ μg/mL for detecting KRAS. The sensor with azulene dye A1 and TiO_2_-Pt/reduced graphene oxide had a lower limit of quantification of 2.64 × 10^−7^ μg/mL KRAS. These sensors were selective for KRAS with minimal interference from other substances. The sensors were used to detect KRAS in whole blood samples from colon cancer patients and results correlated well with KRAS detection in tumor tissue samples. The electrochemical sensors show promise as sensitive and selective tools for the minimally invasive detection of the colon cancer biomarker KRAS. Shu et al. [145] fabricated a biosensor that uses graphene as the electrode material, along with a newly synthesized DNA marker called biferrocene to amplify the detection signal. It also utilizes the enzyme exonuclease III to repeatedly cleave the target DNA and recycle it, further amplifying the signal. The combination of graphene’s electrical conductivity, biferrocene’s two ferrocene redox units, and exonuclease III’s digestion of target DNA strands enables ultrasensitive detection down to 20.4 fM. The biosensor was highly selective for fully complementary DNA versus mismatched DNA. Overall, this novel homogeneous electrochemical biosensor allows for the rapid, simple, low-cost, and highly sensitive detection of KRAS mutations without complex probe immobilization or labeling steps. Wang et al. [146] reported an electrochemical DNA biosensor for detecting mutations in the KRAS gene. The biosensor uses a supersandwich structure on a modified graphene and AuNPs electrode to amplify the detection signal. In the presence of target KRAS DNA, a complex is formed with a capture probe, signal probe, and auxiliary probe, creating a long concatamer that contains many copies of the signal molecule methylene blue. This greatly amplifies the detection signal. The biosensor exhibited excellent sensitivity, with a detection limit of 35 aM for target KRAS DNA. It also demonstrated good selectivity in distinguishing complementary, single-base mismatched, and non-complementary DNA sequences. The biosensor showed a linear detection range of 0.1 fM to 0.1 μM for target KRAS DNA.

### 4.4. The Detection of miRNA Cancer Biomarkers

**Figure 10:**
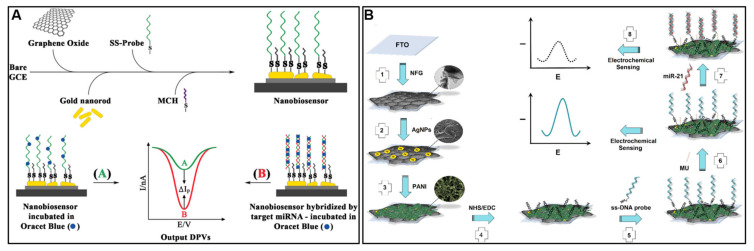
(**A**) Illustration of the assembling and working procedure of the proposed electrochemical biosensor for miR-155 detection. (**B**) Fabrication of nanocomposite/ss-DNA probe/MU/target miRNA for miRNA-21 detection. Reproduced with permission [148,149].

MicroRNAs are short, non-coding RNAs that regulate gene expression and serve as promising biomarkers for cancer diagnosis and prognosis. Pothipor et al. [147] designed an electrochemical biosensor for the simultaneous detection of three microRNA biomarkers related to breast cancer—miRNA-21, miRNA-155, and miRNA-210. The biosensor utilizes a composite film of AuNPs, graphene quantum dots, and GO (AuNPs/GQDs/GO) deposited on a three-electrode, screen-printed carbon array, which provides high surface area and excellent electron transfer capability. Capture probes specific to each miRNA target are immobilized on the electrodes, and hybridization with target miRNAs causes decreased electrochemical signals of redox indicators (anthraquinone, methylene blue, polydopamine) at each electrode. The sensor demonstrated a wide linear detection range of 0.001–1000 pM and low limits of detection down to 0.04 fM for miRNA-21. It demonstrated high selectivity against mismatched sequences, reproducibility between electrodes, and stability over 3 weeks of storage. The sensor was successfully applied to the simultaneous quantification of the three miRNA targets spiked into diluted human serum, with recoveries of 98.5–112%. Azimzadeh et al. [148] used an oligo-hybridization strategy for the detection of miRNA-155. The sensor is based on a glassy carbon electrode modified with GO and gold nanorods, providing a high surface area for sensitivity. Thiolated single-stranded DNA probes designed to capture miRNA-155 are attached to the surface. The sensor utilizes a new electroactive label, Oracet Blue, which intercalates into the double-stranded DNA-RNA hybrids formed after miRNA capture (Figure 10A). This greatly enhances detection sensitivity. The sensor demonstrated a wide linear detection range of 2 fM to 8 pM and an ultra-low limit of detection of 0.6 fM miRNA-155. It also showed excellent selectivity, able to discriminate between fully complementary targets and sequences with just one or three base mismatches. The direct detection of miRNA-155 spiked into human plasma samples was achieved with high recovery and reproducibility. Similarly, Salahandish et al. [149] reported an electrochemical biosensor for the detection of miRNA-21. The sensor uses a nanocomposite coating made of N-doped graphene, AgNPs, and PANI on an FTO. This coating increases the electrode’s surface area and conductivity to enhance sensitivity. The electrode is then functionalized with a complementary DNA probe to capture the target miRNA-21 (Figure 10B). Using differential pulse voltammetry and an optimized set of parameters, the sensor demonstrated a wide detection range of 10 fM to 10 μM, a low limit of detection of 0.2 fM, and a good sensitivity of 2.5 μA/cm^2^. Testing in spiked blood samples showed good selectivity and recovery. Table 4 details recent graphene-based electrochemical sensors for miRNA detection.

## 5. Challenges and Future Directions

While graphene electrochemical biosensors show potential for sensitive and selective cancer diagnosis, there remain significant challenges to translating this emerging technology into widespread clinical adoption. Ongoing research efforts aim to address these challenges and open up new opportunities to further improve analytical performance and real-world applicability.

One key challenge is regarding problems with the reproducibility and reliability of sensor fabrication and performance. Graphene synthesis methods, functionalization strategies, immobilization chemistries, and nanomaterial assembly introduce variability in the final sensor architecture and behavior. This leads to sensor-to-sensor disparities in sensitivity, selectivity, linear range, and limits of detection during cancer biomarker analysis. Establishing standardized fabrication protocols and quality control measures will be essential. Automation using microfluidics and lab-on-a-chip approaches could aid large-scale manufacturing. The systematic optimization of surface functionalization and nanomaterial integration can tailor sensor designs for specific cancer biomarkers to improve reliability across sensor batches.

Another major obstacle is the poor stability and shelf life of current graphene electrochemical biosensors. Biomolecule immobilization and the subsequent target binding introduce conformational changes in receptors like antibodies and aptamers over time, reducing their binding affinity. The numerous nanomaterial interfaces are also susceptible to degradation. This impacts the long-term storage ability and reproducibility of sensors. Stabilization strategies including crosslinking approaches for biorecognition elements and the addition of protective polymers or surfactants for nanomaterials need investigation. Developing calibration and blocking protocols to mitigate activity loss prior to sensor use would enhance robustness.

Selectivity against the complex milieu of non-specific proteins, biomarkers, and cells present in real patient samples poses a further key challenge. Novel selective membranes, hydrogels, and polymers that prevent fouling while allowing for the diffusion of specific targets to the sensor require exploration. Graphene itself can be doped with atoms like nitrogen to improve its hydrophilicity and antifouling properties. Advanced surface functionalization for greater target specificity, combined with optimized sensor geometries maximizing target capture, can enhance selectivity. The use of auxiliary reagents and data processing to filter non-specific signals can augment performance in biological fluids. Expanding testing with diverse patient samples rather than spiked artificial samples will clarify clinical viability.

Most reported electrochemical biosensors use planar 2D graphene electrodes, which limit sensitivity due to their modest surface area. Scalability also remains constrained. Moving to 3D porous graphene networks, composites, and foams provides greater surface area for enhanced biomarker capture and signal transduction. However, optimizing synthesis conditions for reproducible 3D nanostructures and preventing biomolecule leaching from the pores requires work. Free-standing 3D graphene materials allow ease of integration with existing electrode substrates. Mass producible designs leveraging screen printing show promise for commercial translation.

Integration with microfluidics and lab-on-a-chip technology can allow automated sample processing, biomolecule separation, target preconcentration and multistep analysis workflows to further augment graphene sensor performance and practicality. Multiplexing capabilities can be enhanced by fabricating sensor arrays tailored to different cancer biomarkers. Coupling with smartphone connectivity and readout circuits will enable point-of-care operation. Patient studies to validate clinical accuracy against established methods like ELISA and PCR in identifying early-stage cancers and monitoring treatment are vital. Regulatory approval will be contingent on demonstrations of reliability, safety, efficacy, and health outcomes relative to standard of care. An emerging opportunity is the integration of graphene with well-established silicon-based semiconductor technologies for leveraging their complementary advantages. For example, graphene can be integrated with silicon FETs through techniques like the CVD growth of graphene on silicon. This allows graphene’s high sensitivity to be combined with silicon’s ease of manufacturing and integration with electronics. Hybrid graphene-silicon biosensors could potentially offer scalable fabrication along with the high performance of graphene for point-of-care diagnostic devices.

Expanding the breadth of targets beyond common protein antigens and DNA mutations presents a rich opportunity area. The graphene platform offers versatility for the detection of many emerging cancer biomarkers like exosomes, circulating tumor cells, metabolites, and glycoproteins. Explorations into novel biorecognition elements beyond antibodies and DNA probes can impart greater affinity, specificity, and multiplexing capabilities. The hybridization of biological molecules with synthetic reagents through bioconjugation chemistries can augment performance.

Harnessing the full scope of electrochemical techniques beyond amperometry, such as impedimetric, potentiometric, and conductometric transduction, can reveal deeper insights into target binding mechanisms and cancer states. The modification of electrode interfaces with novel nanomaterials to impart enzyme-mimicking properties for enhanced electrocatalysis warrants study. Extending lab-based research to animal models and clinical settings will accelerate translation towards practical cancer diagnostics, theranostics, and point-of-care testing.

Extensive clinical validation with large patient sample sizes is essential to establish sensitivity and selectivity in real patient samples, which may behave differently from spiked samples in buffer solutions. Stringent quality control and testing is critical prior to regulatory approval for clinical use. Furthermore, toxicity concerns with certain forms of graphene need to be addressed to ensure biocompatibility.

Realizing the immense potential of graphene electrochemical biosensors for sensitive, rapid, and low-cost cancer diagnosis faces ongoing challenges in reproducibility, stability, selectivity, scalability, integration, validation, and commercialization. However concerted research efforts to address these challenges and expand capabilities promise continued exciting progress, accelerating their clinical implementation. Ongoing developments in advanced nanomaterial design, surface functionalization, electrochemical techniques, microfluidic integration, and biorecognition element innovation in conjunction with expanded clinical testing will usher these flexible and powerful platforms into mainstream use for early cancer detection, guiding therapeutic interventions and improving patient outcomes.

## 6. Conclusions

In summary, graphene-based electrochemical biosensors have emerged as promising platforms for the sensitive, selective, and rapid diagnosis of cancer. The exceptional properties of graphene, including high surface area, excellent conductivity, ease of functionalization, and superior electrocatalytic activity, enable the construction of sensors with improved performance compared to traditional electrode materials. The ability to efficiently immobilize a variety of biorecognition elements like antibodies, aptamers, peptides, and DNA/RNA probes imparts specificity towards cancer biomarkers. Additionally, graphene composites with nanomaterials, 3D porous architectures, and microfluidic integration further augment analytical capabilities and practical functionality.

Significant advances have occurred in developing graphene electrochemical biosensors for detecting key cancer biomarkers such as proteins, circulating tumor cells, DNA mutations, miRNAs, and metabolites. Innovative sensor designs, immobilization strategies, and signal enhancement approaches have enabled detection limits down to femtomolar and even attomolar levels, alongside high selectivity. Testing with clinical samples shows promise for early diagnosis and point-of-care assessment. However, challenges remain including reliability, reproducibility, stability, scaling up, selectivity in complex media, system integration, validation, and regulatory approval.

Ongoing research to address these challenges, while expanding the range of detectable biomarkers and electrochemical techniques, continues to push graphene biosensor technology forward. Their flexibility, sensitivity, simplicity, and affordability make them well-suited for transitioning cancer diagnostics from centralized labs towards widespread point-of-care clinical use. The standardization of fabrication protocols and quality control measures will aid manufacture. The optimization of surface chemistries, advanced nanocomposite materials, 3D architectures, microfluidic integration, and expanded multiplexing capabilities can further improve performance and practicality. Wider clinical testing and demonstrations of health outcomes versus standard methods are critical to regulatory approval and healthcare adoption. Overall, graphene electrochemical biosensors promise continued exciting advances in coming years, helping to enable early diagnosis, cancer screening, and personalized therapy monitoring to improve patient outcomes.

## Figures and Tables

**Table 1:** Recent graphene-based electrochemical sensors for PSA detection.

PSA Electrochemical Sensor	Linear Detection	LOD	Reference
Peptide and GO/AgNPs	5 pg/mL to 20 ng/mL	0.33 pg/mL	[115]
H-Gr/PdNPs	0.025 to 204.8 ng/mL	8 pg/mL	[116]
AuNPs/rGO/THI-aptamer	0.05 to 200 ng/mL	10 pg/mL	[30]
GC/AuNPs/rGOAuNPs/anti-PSA	1 to 36 ng/mL	2 pg/mL	[117]
GCE/rGO/Au NPs/Ab1 with redox probes@Ab2/Si@Au NPs nanocomposites as labels	0.01 to 25 ng/mL	0.004 ng/mL	[116]
Ab/GA/CS-rGO/ITO	1 to 5 ng/mL	0.8 pg/mL	[118]
3D GF/CS-Au NPs	0.001 to 200 ng/mL	0.51 pg/mL	[119]
FTO/Au CC-NRs/rGO (composite)/CS	0.1 to 150 ng/mL	0.016 ng/mL	[120]
rGO/Ag Nanoparticles	1 to 1000 ng/mL	0.01 ng/mL	[121]
Graphene–Au NPs	1 to 10 ng/mL	0.59 ng/mL	[122]

**Table 2:** Recent graphene-based electrochemical sensors for CEA detection.

CEA Electrochemical Sensor	Linear Detection	LOD	Reference
CEA/BSA/anti-CEA/PtPd/N-GQDs@Au/GCE	5.0 fg/mL to 50.0 ng/mL	2.0 fg/mL	[124]
Anti-CEA/PBSE/graphene/Cu	1.0 to 25.0 ng/mL	0.23 ng/mL	[47]
GCE/AgPt NRs-rGO/Ab/BSA/CEA	5.0 fg/mL to 50.0 ng/mL	1.43 fg/mL	[125]
Anti-CEA/rGO-PtAu NPs/GCE	10.0 to 1.0 × 10^8^ fg/mL	7.0 fg/mL	[126]
Anti-CEA/3D-rGO-MWCNTs/Ag–Au NPs/GCE	0.0001 to 80.0 ng/mL	3.0 pg/mL	[127]
Anti-CEA/PEDOT/Ag@BSA/rGO/CNTs-COOH/Au	0.002 to 50.0 ng/mL	0.1 pg/mL	[123]
Anti-CEA/rGO/MoS_2_@PANI/GCE	0.001 to 80.0 ng/mL	0.3 pg/mL	[128]
CEA-Ab/GZ-PYSE/SPCE	0.01 to 10.0 ng/mL	0.004 ng/mL	[129]
Ab_2_-CZTS/CEA/BSA/Ab_1_/GN-Au/GCE	5.0 × 10^−4^ to 20.0 ng/mL	0.15 pg/mL	[130]
CEA/BSA/Ab_1_/3DPt/HGO-MGCE	0.001 to 150.0 ng/mL	0.0006 ng/mL	[31]

**Table 3:** Recent graphene-based electrochemical sensors for CTCs detection.

CTCs Electrochemical Sensor	Linear Detection	LOD	Reference
Aptamer/electroactive species-loaded AuNP probes	5 to 500 cells/mL	4 cells/mL for Ramos cells 3 cells/mL for CCRF-CEM cells	[39]
MCF-7/S1/Gr/AuNPs/GCE	50 to 1 × 10^4^ cells/mL	27 cells/mL	[134]
MPA/Rh-NPs/rGONs/graphite electrode	5.0 to 1.0 × 10^5^ cells/mL	1 cells/mL	[133]
Fe_3_O_4_NPs/rGO/MoS_2_	15 to 45 cells/mL	6 cells/mL	[135]
FA-GAM-OA	5.0 to 1.0 × 10^6^ cells/mL	5 cells/mL	[84]
A/Glu-GQD-Pd@Au	3.0 to 1.0 × 10^6^ cells/mL	2 cells/mL	[80]
Herceptin-conjugated graphene	1 to 80 cells/mL	-	[136]
Apta-PGO-LAPS’	5 to 5000 cells/mL	-	[137]
MB-aptamers/dsDNA/AuNP-modified GE	1 × 10^2^ to 1 × 10^6^ cells/mL	23 cells/mL	[138]
HRP-Si/AuNPs-Ab2/MCF7/Ab1-SA/GO/microchip	1 to 10^5^ cells/mL	10 cells/mL	[139]

**Table 4:** Recent graphene-based electrochemical sensors for miRNA detection.

miRNA Electrochemical Sensor	Analyte	Linear Detection	LOD	Reference
AuNPs/GQDs/GO/SPCE	miRNA-21, miRNA-155, miRNA-210	0.001 to 1000 pM	0.04 fM, 0.33 fM, 0.28 fM	[147]
SS-probe/GO/GNR/GCE.	miRNA-155	2.0 fM to 8.0 pM	0.6 fM	[148]
miRNA-21/MCH/capture DNA-21 probe/dyes-AuNPs-coated nanocomposite-modified 2SPCE	miRNA-21	0 to 1000 pM	1.2 fM	[150]
nanocomposite/ss-DNA probe/MU	miRNA-21	10 fM to 10 µM	0.2 fM	[149]
anti-miRNA/GA/AGr/GCE	miRNA-155	30 fM to 1 pM	12.5 fM	[151]
PHSGNPs/metal ion/cap-DNA/miRNA	miRNA-155, miRNA-21, miRNA-16	1 fM to 10 nM	0.98 fM, 3.58 fM, 0.25 fM	[152]
GO/GNR/miR	miR199a-5p	15 fM to 148 pM	4.5 fM	[153]
GO/Au NPs	miRNA-155	1 nM to 10 fM	3.3 fM	[154]
GO	miR-141	1 nM to 1 fM	5 fM	[155]
AuNPs/perovskite/GO	miRNA-21	10 fM to 100 nM	2.94 fM	[156]

## Data Availability

Data sharing not applicable.
